# Internally directed cognition and mindfulness: an integrative perspective derived from predictive and reactive control systems theory

**DOI:** 10.3389/fpsyg.2014.00429

**Published:** 2014-05-20

**Authors:** Mattie Tops, Maarten A. S. Boksem, Markus Quirin, Hans IJzerman, Sander L. Koole

**Affiliations:** ^1^Department of Clinical Psychology, VU University AmsterdamAmsterdam, Netherlands; ^2^Rotterdam School of Management, Erasmus UniversityRotterdam, Netherlands; ^3^Donders Institute for Brain, Cognition and Behaviour, Centre for Cognitive NeuroimagingNijmegen, Netherlands; ^4^Institute of Psychology, University of OsnabrückOsnabrück, Germany; ^5^Tilburg School of Behavioral and Social Sciences, Tilburg UniversityTilburg, Netherlands

**Keywords:** mindfulness, default mode network, insula, imagery, rumination, observer perspective, self-reflection, prospection

## Abstract

In the present paper, we will apply the predictive and reactive control systems (PARCS) theory as a framework that integrates competing theories of neural substrates of awareness by describing the “default mode network” (DMN) and anterior insula (AI) as parts of two different behavioral and homeostatic control systems. The DMN, a network that becomes active at rest when there is no external stimulation or task to perform, has been implicated in self-reflective awareness and prospection. By contrast, the AI is associated with awareness and task-related attention. This has led to competing theories stressing the role of the DMN in self-awareness vs. the role of interoceptive and emotional information integration in the AI in awareness of the emotional moment. In PARCS, the respective functions of the DMN and AI in a specific control system explains their association with different qualities of awareness, and how mental states can shift from one state (e.g., prospective self-reflection) to the other (e.g., awareness of the emotional moment) depending on the relative dominance of control systems. These shifts between reactive and predictive control are part of processes that enable the intake of novel information, integration of this novel information within existing knowledge structures, and the creation of a continuous personal context in which novel information can be integrated and understood. As such, PARCS can explain key characteristics of mental states, such as their temporal and spatial focus (e.g., a focus on the here and now vs. the future; a first person vs. a third person perspective). PARCS further relates mental states to brain states and functions, such as activation of the DMN or hemispheric asymmetry in frontal cortical functions. Together, PARCS deepens the understanding of a broad range of mental states, including mindfulness, mind wandering, rumination, autobiographical memory, imagery, and the experience of self.

## INTRODUCTION

People’s mental lives shift from moment to moment between states that differ in the level and quality of awareness of inner experience or the external world. Some mental experiences may be occupied by current events in the external environment, yet others by contents from episodic memory. But how and when does one mental state give way to another? And do internally directed mental states and shifts toward externally directed mental states have relevant functions? What exactly discriminates mental states that appear overlapping (e.g., ruminative vs. other kinds of mind wandering)? We think that to answer these questions psychologists require an integrative account on the basis of relevant brain mechanisms. In the present paper, we seek such an integrative account, and suggest that shifts between mental states, as well as characteristics of those mental states, represent workings of only a small set of control systems in the brain that control behavior, cognition, homeostasis, and emotion.

But how can we understand internally directed mental states? Traditionally, researchers have not paid much attention to these states, as most research has been directed to the workings of *externally* directed mental states and cognitive functions through the performance of a number of different tasks. In contrast to this typical approach, some researchers have recently started investigating neural networks during resting states, and especially what has been dubbed the “default mode network” (DMN; Raichle et al., 2001). The DMN becomes active at rest in case no external stimulation – such as a cognitive task offered in a typical study – is provided to perform, and may be implicated in internally directed and self-reflective cognition. In contrast to this putative association with self-awareness, activity in this area is inversely correlated in functional-imaging studies with the activation in the anterior insula (AI) that is associated with awareness and task-related attention (Craig, 2009). The inverse correlation between the DMN and the AI has led to competing theories of the neural substrates of something of direct relevance to internally directed mental states, awareness, stressing either the role of the DMN in self-awareness (Boly et al., 2008; Greicius et al., 2008; Vanhaudenhuyse et al., 2010) or the role of interoceptive and emotional information integration in the AI in subjective feeling and awareness of the emotional moment (Craig, 2009). We are not aware of previous theoretical attempts to integrate these different aspects or qualities of awareness in one framework.

In the present paper, we will apply the theory of predictive and reactive control systems (PARCS; Tops et al., 2010) to suggest that the DMN and AI each belong to one of two different behavioral and homeostatic control systems, and we will explain this guided by our knowledge on internally directed mental states. The respective functions of the DMN and AI in a specific control system explains their association with different mental states or qualities of awareness, while it also explains how mental states can shift from one state to another depending on the relative dominance of one control system or interactions between the systems.

We think that PARCS is relevant for explaining internally directed mental states, as the theory is based on fundamental differences in the way novel information is processed compared to familiar information, the relevance of which we explain below. In particular we focus on how cognition and behavior are controlled in novel or unpredictable circumstances compared to familiar and predictable circumstances. As outlined in earlier work (Tops et al., 2010, 2014a), the brain relies on reactive control systems applying feedback-guided control for handling novelty. By contrast, in familiar circumstances the brain relies on predictive control guided by internal models shaped during previous learning. In the present paper, we aim to explicate how internally (compared to externally) directed mental states derive from these reactive or predictive control systems, and how shifts between internally and externally directed mental states are explained by shifts between reactive and predictive control. For instance, learning a novel but predictable task first requires involvement of the reactive control system for handling novelty, but, after the forming of internal working models, the predictive system will take over and control will become more habitual, allowing for internally directed mental states, often expressed in people’s wandering of their own mind.

In Section “The Theory of Predictive and Reactive Control Systems,” we summarize the main tenets of PARCS. This description will include an integration of DMN’s functions into the predictive system. In forming a coherent framework of a sense of self, we further discuss the crucial role of the AI in the reactive control system. In so doing, we partly offer a reinterpretation of Craig’s (2009) theory of interoceptive awareness. We will also discuss the role of left hemisphere areas in “translating” between reactive and predictive control (i.e., in assimilating novel information to preexisting internal models). In exploring these links, we further elaborate upon PARCS than was done previously (e.g., Tops et al., 2010). In Section “The Dynamics of Reactive and Predictive Control Systems in Internally Directed Cognition and Mindfulness.” we explain how PARCS predicts and explains characteristics of internally directed cognition, such as in rumination, mind wandering, experience of self, prospection, and the field and observer perspectives in imagery and autobiographical memory. We apply those characteristics, such as temporal and spatial focus of awareness, to help understand the state of mindfulness and the processes that lead to getting into a mindful state, a topic that has been captured the recent attention of many researchers. The discussion of mindfulness in Section “Accepting without Judgment” is therefore a way to synergize externally and internally directed mental states. In Section “Discussion,” we consider our main conclusions, alongside PARCS’ broader implications for understanding and studying internally directed cognition.

## THE THEORY OF PREDICTIVE AND REACTIVE CONTROL SYSTEMS

### SYSTEMS FOR REACTIVE vs. PREDICTIVE CONTROL

Before we discuss how the DMN functions as part of a larger overarching system, let us turn to the basic underlying structures that enable mammals to deal with their environment. Support has been accumulating that mammalian brain systems contain two cortical systems controlling behavior, a reactive and a predictive control system (reviewed in Tucker and Luu, 2007; Tops et al., 2010). An anterior temporal–ventrolateral prefrontal cortical [including the inferior frontal gyrus (IFG), AI, anterior hippocampal formation, ventral striatum, and amygdala] system is specialized in processing novel, salient, and urgent stimuli and in reactive (e.g., feedback-guided) control of behavior in unpredictable environments. Reactive control thus represents a specialized mode of operation for detecting new information, encoding it in memory and assimilating it into preexisting internal models, thus facilitating future control by the predictive system (Hasher and Zacks, 1979; Tops and Boksem, 2011a). By contrast, a posterior medial–dorsal cortical system [including the posterior cingulate cortex (PCC) and precuneus] processes familiar stimuli and controls behavior in predictable environments, guided by predictive internal models, including models of others and self. In effect, these models include, but are not limited to, what attachment theorists have called “internal working models” (Bowlby, 1988), a topic we will venture into later. Prediction and internal models in the dorsal system allow for feedforward and partly automated action control, thereby reducing the necessity to devote attention to external cues. Increased feedforward control means that a greater amount of action steps are programed and performed in a fluent sequence, without waiting for feedback between each step.

The reactive guidance by momentary environmental stimuli is associated with attentional focus on stimuli that are urgent and close in time and space. Those stimuli can be positive (“I have to catch that reward that is in my reach before it gets away”) or negative (“I have to get away from that danger before it gets me, because I’m in its reach”). The reactive control system is involved in, and relates stimuli to, the experienced self in the here and now. By contrast, there is typically less urgency and focus on the moment (i.e., broader, more global focus in time and space) when behavior is guided predictively (in feedforward fashion) by internal models. The characteristics of reactive and predictive control are discussed in more detail elsewhere (Tops et al., 2010, 2013a,b; Tops and Boksem, 2011a, 2012). We summarized them here in **Table 1**.

**Table 1:** Characteristics of the reactive and predictive control systems.

	Reactive system	Predictive system
Origin	Paleocortical	Archicortical
Visceral functional base	Viscerosensory	Visceromotor
Cortical network	Ventrolateral	Dorsomedial
Central areas	IFG, AI, temporo-parietal junction, anterior hippocampal formation, ventral striatum, amygdala	PCC, precuneus, angular gyrus, parahippocampal cortex, posterior hippocampal formation, mPFC, DLPFC
Environment/situation	Low-predictable, changing	High-predictable, stable
Triggering stimuli	Novelty	Familiarity
Cognitive mode	Object/context free	Configural/internal model
Motor control	Feedback/reactive	Feedforward/predictive
Learning stage	Early	Late
Attention	Focused/local	Global
Temporal focus	Immediate, momentary, urgent, delay discounting	Prospection/extended
Emotional intensity	High	Low

*IFG, inferior frontal gyrus; AI, anterior insula; PCC, posterior cingulate cortex; mPFC, medial prefrontal cortex; DLPFC, dorsolateral prefrontal cortex.*

It is important to note that PARCS is *not* built around the concepts reactive and predictive. Rather, we use the terms reactive and predictive as labels to refer to functional systems that combine a set of features that together are optimal in controlling behavior and physiology in unpredictable or predictable circumstances. For example, some features of the reactive system do not fit at all to the label reactive (e.g., sustained attentional control or active maintenance of task goals by the reactive system). Moreover, each system contains certain features that are evolutionarily older and seem more primitive (e.g., related to reactivity, impulsivity, stimulus controlled) while other features of the same system are evolutionarily more recent and appear at a higher (e.g., cognitive control, reflective) level. Predictive control by internal models – and the chances of focusing one’s mental state inward – may have evolved later and at first glance appear more “sophisticated” than reactive control. However, the point we want to make is that both systems are continuing to evolve and have *both* developed higher-level control. In the next section, we further describe and illustrate the development of higher-level control in the predictive and reactive systems by taking the DMN and AI as respective examples of those larger systems. In turn, in Section “The Dynamics of Reactive and Predictive Control Systems in Internally Directed Cognition and Mindfulness,” we apply our distinction between reactive and predictive control to explicate the differing characteristics of internally directed cognition across different contexts.

### THE DEFAULT MODE NETWORK AS PART OF THE PREDICTIVE SYSTEM

The DMN has recently been associated with activity in case no external stimulation was offered to participants, but the literature has suggested functions beyond inactivity. PARCS suggests the DMN (together with the dorsal executive network) – beyond being involved with such inactive states – to be part of a predictive control system. There are a number of converging lines of research supporting this view. First, in contrast to the reactive system that incorporates fast associative learning, the predictive system is specialized in guiding behavior by internal models that are formed in long-term memory and kept stable by slow learning of the environment’s predictability. The DMN consists of brain areas involved in the predictive system, including in the PCC, precuneus, angular gyrus, medial temporal lobe, and medial prefrontal cortex (mPFC; Raichle et al., 2001). Second, beyond activity in case of no external stimulation, this network also becomes active when individuals engage in internally focused tasks including autobiographical memory retrieval, envisioning the future, long-term script processing and conceiving the perspectives of others (Ruby et al., 2002; Buckner and Carroll, 2007; Jack et al., 2012), which is consistent with the role of the dorsal system in predictive behavioral control. Other parts of the predictive control system are the dorsal executive network that consists of the dorsolateral prefrontal cortex (DLPFC; Seeley et al., 2007) and the dorsal attentional system (Shulman et al., 2009). The areas of the DMN are connected to the dorsolateral striatum, anterior thalamus, and DLPFC (see Tucker and Luu, 2007; Tops et al., 2010).

The functions typically ascribed to the DMN run parallel to functions in behavior control by internal models that we described earlier to be part of PARCS’ predictive system (Tops et al., 2010, 2013a, 2014a; cf. Tucker and Luu, 2007). Much similar, Buckner and Carroll (2007) have suggested the DMN to support the performance of internal mentation. This network may provide a platform for putting together dynamic mental models and scenarios that are largely detached from the specific or current external world, all abilities relevant for focusing one’s attention inward. In previous work, we have referred to these mental models and scenarios as “context models” (cf. Tucker and Luu, 2007) or “internal (working) models.” Typically, DMN’s functioning has been described to contain elements of autobiographical episodic memory and self-related prospective thoughts. Also, researchers have suggested that continuously ongoing internally directed processes through the DMN function as “simulator” and/or predictor of future events, building upon previous experiences, using past experiences and internal working models to plan for the future (Buckner and Carroll, 2007; Tucker and Luu, 2007; Tops et al., 2010; Smallwood et al., 2013a). One could summarize these three points by stating that the DMN’s main function is that of predictive control.

This concept of the internal model – which truly is the basis for our idea of predictive control – originates in the work of the philosopher Kenneth Craik. Craik (1943) noted the adaptiveness of the ability of thought to plan for future events. He stressed the survival value of and natural selection for the ability to plan for future events. According to Craik, prediction occurs when a “small-scale model” consisting of brain events is used to represent not only the external environment, but also the individual’s own possible actions. Internal models thus allow people to mentally experiment with alternative realities, using past knowledge to respond to the present and future. One of the most notable examples to apply Craik’s ideas to social relationships and attachment was of course attachment theorist John Bowlby (Bowlby, 1988), who was followed up more recently by other psychologists applying the concept of the mental model to perception, cognition, personality, and therapy (Kelly, 1955; Piaget, 1960; Johnson-Laird, 1983; Clark, 2013a,b; Hirsh et al., 2013; Hassabis et al., 2013).

The predictive system as we have described it in PARCS largely overlaps with a recent incarnation of these internal models, focused on a “situation model,” an internal model, that represents relationships between entities, motivations, actions, and outcomes (Ranganath and Ritchey, 2012). Specifically, a situation model is like a schema that specifies the gist of the spatial, temporal and causal relationships that apply within a particular context, and relies on the same neural correlates of the dorsal predictive system. Behavioral research suggests that situation models support a diverse range of cognitive functions that are relevant for planning for future events, such as language comprehension, inductive reasoning, decision making, learning of cause–effect relationships, and a more general social cognition (Zwaan and Radvansky, 1998).

Consistent with a role for the DMN to simulate and predict, the default and the dorsal executive network can closely cooperate in supporting thought processes, and seem to do so at times when primary sensory cortices discontinue the processing of external perceptual information. For example, while viewing videos, cortical activity momentarily decreased during eye blinks in the dorsal executive network but increases in the DMN (Nakano et al., 2013). When the video was removed from the screen, there were no reciprocal changes in brain networks, suggesting that increased activity in the DMN during eye blinks reflects internal processing. In line with the predictive role of the DMN, activation of this network was associated with faster actions when actions required input from memory but did not depend upon immediate perceptual input, but activation of this network was associated with slower actions when they were based on perceptual input (Smallwood et al., 2013b). In their work, Christoff et al. (2009) find converging support that mind wandering – which is based on internally directed cognition – engaged both the DMN and DLPFC. When participants were engaging in mind wandering, they displayed positive functional connectivity between these regions and negative functional connectivity between the DMN and primary sensory cortices (Christoff, 2012). In this work, this was interpreted as reflecting involvement of mind wandering in simulation and prediction in action control. Aligning with the idea that mind wandering is for planning for future events, goal-directed simulation engaged the DMN and the DLPFC (Gerlach et al., 2011).

Whereas we have now discussed the role of the DMN in internal models and mind wandering, there is also clear support for the role of the DMN in social functioning. Indeed, areas of the DMN (precuneus and dorsomedial prefrontal cortex (PFC)], together with the IFG, are also activated while observing social interactions (Iacoboni et al., 2004) and with increased social working memory load (Meyer et al., 2012). This latter activation we interpret to reflect the recruitment of internal models of normative principles for social relations (cf. Rai and Fiske, 2011). In other words, the DMN is not only activated when there is no externally focused task to perform, but more broadly is implicated when internal models are attended to. Based on these converging lines of research, we tentatively conclude that the DMN plays a major part in the dorsal predictive control system.

### THE ANTERIOR INSULA AS PART OF THE REACTIVE SYSTEM

For this section, we select the AI to discuss characteristics of reactive control. The AI is not only an important area of the reactive control system, but like the DMN, it has also been implicated in awareness (Craig, 2009). Beyond that we set the stage for interpreting the AI in terms of PARCS in relation to earlier interpretations, discussing the role of the AI and relating this to the role of the DMN in PARCS will help us to contrast and typify reactive and predictive control effects on awareness and internally directed cognition in Section “The Dynamics of Reactive and Predictive Control Systems in Internally Directed Cognition and Mindfulness.”

In PARCS, the AI is not only a central hub for the reactive system, but also for shifts between reactive and predictive control. Especially the right hemisphere AI and IFG are implicated in the coordination of responses in situations of emergency, novelty, and unpredictability. In unpredictable or novel environments and situations, there are no relevant or effective predictive internal models available. Instead, the reactive system initiates feedback-guided control of behavior. This system controls responding to novel, salient and urgent stimuli and focuses attention on stimuli that are urgent and close in time and space.

Functional and connectional gradients in brain areas including the insula point to the importance of discriminating reactive (ventral) from predictive (dorsal) controls in the brain. Prevailing theories hold that, especially in the right hemisphere, the insula is functionally organized along its caudal–dorsal to rostral–ventral axis, with posterior regions showing more differentiation between interoceptive information of different origins and anterior regions forming integrated representations and awareness of global interoceptive and emotional state and stimulus significance (e.g., Craig, 2009). Recently, support has accumulated that the anterior-ventral insular regions are associated with areas that form the ventral, reactive control system in PARCS. In terms of function the AI forms part of a key emotional appraisal, intensity of social-emotional experience or arousal, and cognitive control network. By contrast, the dorsal posterior insula and dorsal-middle insular regions have been associated with areas that form the dorsal, predictive control system in PARCS, and are activated during functions of predictive control such as sensorimotor integration, skeletomotor body orientation, interoception, and awareness. This support was obtained from meta-analyses (Kurth et al., 2010; Cauda et al., 2012), studies of somatotopic anatomic connections (e.g., Chikama et al., 1997), task-related (e.g., Cauda et al., 2012) and resting state functional connectivity (e.g., Postuma and Dagher, 2006; Taylor et al., 2009; Touroutoglou et al., 2012), and probabilistic tractography (e.g., Cerliani et al., 2012).

Predictive and reactive control systems puts the functional, connectional, and cytoarchitectonically gradients in the insula in a different light, by functionally implicating them in shifts between reactive and predictive control. The rostral-to-caudal functional gradient appears to be similar to, and interconnected with, a rostral-to-caudal functional gradient from the AI through mPFC and the anterior cingulate cortex (ACC) to the PCC (see Tops and Boksem, 2012). The rostral–ventral to caudal–dorsal gradient in mPFC displays a functional shift from responding to novel events that trigger “manual” (i.e., momentary feedback guided) control and learning, via feedforward control learning to action selection aspects of more automated action control. This gradient mirrors the pattern of interconnections between cortex and striatum, as IFG/AI and rostral–ventral mPFC are connected to ventral striatum and PCC/precuneus are connected with dorsal striatum. Reentrant loops through the ventral striatum terminate in regions of PFC that are more dorsal than where they begin, forming ventral (limbic), central (associative), and dorsal (motor) corticostriatal loops through which information can pass from ventral striatum forward into dorsal striatum, and this shift from ventral to dorsal striatum is associated with a shift from hedonic processing toward automated, non-hedonic habitual action control (Alcaro and Panksepp, 2011). In terms of PARCS, this shift in processing reflects performance learning through the formation of internal models that allow for increased predictive control.

As a central area of the reactive control system, the AI has been implicated in homeostatic regulation (and in sympathetic nervous system activation and interoception; Craig, 2005). Indeed, situations of unpredictability, uncontrollability, and emergency have important consequences for homeostatic control. Consistent with reactive control, right AI activation is associated with high arousal in response to stimuli of the moment or stimuli that are expected at any moment. PARCS suggests that anxious anticipation involves the feeling that one should stay ready in reactive control mode to handle imminent unpredictable or uncontrollable stimuli. Indeed, anxious anticipation has been found to activate the right AI, and more so in trait anxious individuals (Waugh et al., 2008; Lovero et al., 2009; Carlson et al., 2011; Simmons et al., 2011). This may explain why individuals with anxiety disorders exhibit elevated right AI activity (Etkin and Wager, 2007).

**FIGURE 1:**
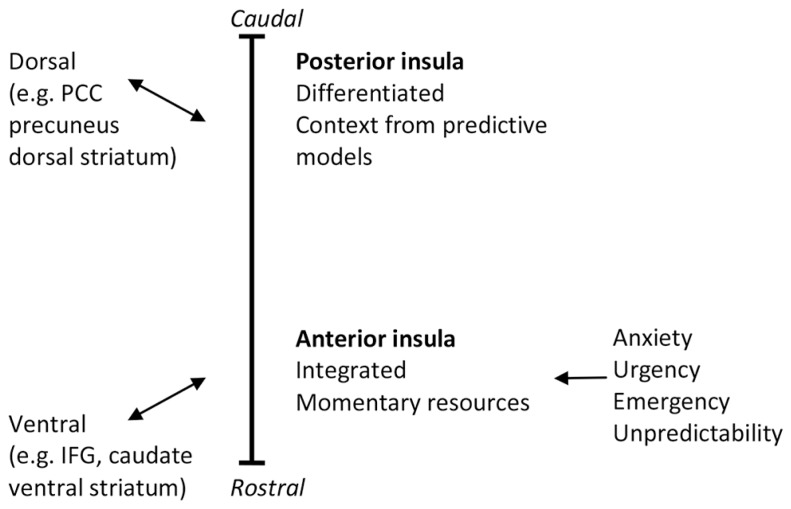
**The caudal–dorsal to rostral–ventral functional and connectivity gradient in the insula.** PCC, posterior cingulate cortex; IFG, inferior frontal gyrus.

By relating the differentiated viscerosensory representations in the posterior insula to the predictive control system, PARCS suggests that activation of these areas relate to increased interoceptive self-observation skills (e.g., during mindfulness, see Mindfulness). By contrast, PARCS predicts that urgency, emergency, and unpredictability activate reactive control and the AI and suppress predictive control (see **Figure 1**). In contrast with Craig’s (2009) thoughts on the role of the AI, we note the association of reactive control with anxiety to explain why anxiety is associated with increased undifferentiated awareness of arousal (physiological activation; Pollatos et al., 2007) but less differentiated awareness of specific somatic states (somatic neglect; Koole et al., 2014).

### APPRAISAL AND REAPPRAISAL IN THE RIGHT AND LEFT INFERIOR FRONTAL GYRUS

In this section, we discuss functional hemispheric asymmetry in the IFG, an area that partially borders to and often coactivates with the AI, and constitutes another sub-area of the reactive control system. We do so to understand processes of appraisal and reappraisal, which attempt to relate novel information and inconsistencies to internal models. After a short description of the role of the right IFG, we will specifically focus on a translational function of the left IFG between reactive and predictive control (i.e., the process of assimilation of novel information to preexisting internal models). This function of networks involving the left IFG produces a third quality of experience and awareness that can be discriminated from awareness produced by networks centered on the DMN or right AI/IFG. In Section “The Dynamics of Reactive and Predictive Control Systems in Internally Directed Cognition and Mindfulness,” we discuss how these three different qualities of awareness can be discriminated subjectively and in research on internally directed cognition and mindfulness.

Unexpected stimuli, like novelty and saliency, activate parallel reactive and predictive system networks, the first for appraising the significance of a stimulus, associated with phasic changes in autonomic arousal and preferential recruitment of the right hemisphere amygdala and IFG, and the second for evaluating and updating the stimulus context, which is not reliant on orienting and involves the temporoparietal junction (supramarginal gyrus) and DLPFC in interaction with the hippocampal/parahippocampal region (Williams et al., 2007). Especially in the right hemisphere, nearby or overlapping areas in the IFG are often coactive and implicated in the different aspects that constitute the first response to novelty and saliency, that is, the orienting response (e.g., Downar et al., 2000). The classic orienting response described by Maltzman and Raskin (1965) and Sokolov (1960, 1963) has been thought to be made up of three subcomponents: motoric or response (behavior) inhibition, bottom-up orienting of attention and attentional capture by novelty, and appraisal including elaborative processing of degraded or perceptually ambiguous stimuli. The right IFG is implicated in these components of the orienting response (see, e.g., Aron et al., 2004; Chikazoe et al., 2007; Shulman et al., 2009; Aron, 2011; Tops and Boksem, 2011a) and other aspects of feedback-guided reactive control and appears to coordinate appraisal of novelty, emotional stimuli, and saliency. Indeed, the IFG/AI responds to negative performance feedback, a stimulus that elicits typical orienting responses such as decreased heart rate (Ullsperger and von Cramon, 2003).

**FIGURE 2:**
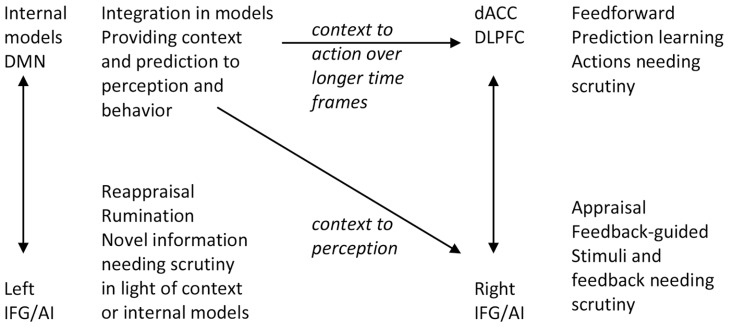
**The functions of left and right IFG are both similar and different.** They take over control and perform scrutiny when there is failure or distraction from feedforward action control (right IFG) or when stimuli are not immediately consistent with context or internal models (left IFG). DMN, default mode network; AI, anterior insula; IFG, inferior frontal gyrus; dACC, dorsal anterior cingulate cortex; DLPFC, dorsolateral prefrontal cortex.

Signals of unpredictability, prediction and performance error, novelty, incongruity, or saliency require individuals to appraise the situation, and signal a need for return from model-guided feedforward control to momentary feedback-guided control (**Figure 2**; Tops and Boksem, 2011b, 2012). Some of those signals may be detected in the IFG/AI that subsequently interrupts predictive control (cf. Tucker et al., 2003; Corbetta et al., 2008; Menon and Uddin, 2010). Briefly observed signals may be processed further through a maintenance working memory function of the IFG (Ranganath et al., 2004; Tops and Boksem, 2011a). If this is followed by reestablishment of predictive motor control by the dorsal ACC, the arousal of the orienting response is allowed to return to baseline. However, besides this process at the level of task performance, habituation of orienting responses to novel, salient, and emotional stimuli often requires *reappraising* the meaning and relevance of the stimulus in relation to higher-level (i.e., self-related) internal models.

One of the ways that allows people to reappraise novel and emotional situations is through their social networks. Culture and social sharing may be important influences that shape or maintain internal models through personal narrative representations (Rimé, 2009; Hirsh et al., 2013). According to a review by Rimé (2009), emotional experiences may occur when prediction fails, when expectations are disconfirmed, and when activities in progress are blocked. They challenge internal models about oneself and the world that are held by individuals to preserve a sense of coherence, predictability, and control. In addition, emotional circumstances generally involve unfamiliar or atypical objects or events that are likely to challenge collective representations and shared knowledge. Given the right conditions under which it occurs, verbal social sharing of emotions and conversation can transform and absorb unfamiliar elements into social internal models and facilitate reappraisal (Rimé, 2009).

The left hemisphere IFG is important in the process of assimilation of novel and emotional information into existing internal models and thereby has an important role in processes in PARCS. Whereas the right IFG is implicated in the *appraisal* of a stimulus, the left IFG is implicated in the *reappraisal* of the stimulus in light of preexisting internal models. Reappraisal can alter emotional responses by changing one’s interpretation of a situation’s meaning. The role of left and right IFG is similar to a large extent; they are involved in appraisal and elaborative processing when more automatic processing of predictive control cannot do the job. However, the left IFG has an important intermediate, translational function between reactive and predictive control systems. The verbalization and semantization functions in the left IFG may have a role in reappraisal processes and verbal sharing of emotions as it helps translating and adapting novel (relatively context-free) reactive emotional experiences into semantic information that can be fitted into internal models (see **Figure 2**).

Functional neuroimaging has revealed that using reappraisal strategies such as reinterpretation of the event in the service of regulating affective responses to emotional stimuli most often activates the left IFG, while strategies to decrease or inhibit appraisal most often activate the right IFG (see Ochsner et al., 2012 for a review). Ochsner et al. (2012) have suggested that this pattern could reflect the differential dependence of reappraisal on linguistic and semantic processes as opposed to attentional processes that generally show a left vs. right hemisphere pattern of relative specialization. Indeed, similar to reappraisal, simply emotion labeling (identifying, naming, and linguistic processing of emotions) activates the left (and right) IFG and decreases arousal and amygdala activity (Torrisi et al., 2013; see Mindfulness). Reappraisal is associated with enhanced memory while emotion suppression is associated with impaired explicit memory of the emotional event; imaging results showed that successful encoding during reappraisal was uniquely associated with greater co-activation of the left IFG, amygdala, and hippocampus, suggesting a possible role for elaborative encoding of negative memories (Hayes et al., 2010). Relatedly, left IFG activation in anticipation of uncontrollable pain related to less avoidance of fitting into internal models, like trait denial coping (e.g., “I pretend that it hasn’t really happened”), and greater attempts at fitting into internal models, like more acceptant coping (e.g., “I learn to live with it”; e.g., Salomons et al., 2007).

Lesion studies support a link of right hemisphere function with cognitive novelty and exploratory behavior, whereas the left hemisphere has been linked to cognitive familiarity and routinization (Goldberg et al., 1994). Based on research on split-brain patients, a left brain interpreter refers to the construction of explanations (in terms of internal models) by the left brain in order to make sense of the world by reconciling novel information with what was known before (Gazzaniga, 2000). The left brain interpreter attempts to rationalize, reason, reappraise, and generalize new information it receives in order to relate the present to internal models formed by the past. Similarly, a right hemisphere appraisal-like mechanism for anomaly or novelty detection has been proposed, vs. a left brain mechanism for maintaining current beliefs (internal models) about the world (Ramachandran, 1995). This may be related to neurophysiological evidence that the left (socially manipulative, action oriented) and the right (emotional perceptions) hemispheres subserve different emotional sets that correspond to “control” and “appraisal” (Henry, 1997). Finally, according to a model by Perlovsky (2011; Perlovsky and Ilin, 2013) the language semantic area in the left IFG guides the development of internal models using narrative information and restrictions on Bayesian processes from culture and collective wisdom that have accumulated in language (cf. Lupyan, 2012; Hirsh et al., 2013).

Resting state functional connectivity in humans between cortical areas and the striatum is consistent with such a central role of the left IFG in the spiraling corticostriatal loops that connect ventral (reactive) with dorsal (predictive) system areas (Di Martino et al., 2008; see The Anterior Insula as Part of the Reactive System). Functional connectivity displayed a gradual transition, in which the ventral striatum was connected to the right IFG (BA 47), the ventral caudate was connected to the bilateral IFG, DLPFC, and ventral ACC, all more extensively in the left compared to right hemisphere. By contrast, the dorsal caudate nucleus was connected to the left IFG (BA 47/45) as well as to the bilateral dorsolateral and dorsomedial PFC and PCC.

The translational role of the left IFG and its language semantic area between reactive and predictive control systems is supported by the tendency of this area in particular to coactivate with both systems and in association with both reactive and predictive control functions. Indeed, in addition to the more obvious frequent coactivations of left and right IFG/AI, a meta-analysis of DMN activation using the activation likelihood estimation (ALE) approach across the domains resting state “default mode,” autobiographical memory, prospection, navigation, and theory of mind, found coactivation of the left IFG (strongest convergence in BA 47) with the DMN (Spreng et al., 2009). Similar results were found in an ALE analysis by Mar (2011)^1^.

There are more specific examples of coactivation of the left IFG with both reactive and predictive systems and control functions. For instance, whereas emotion-introspection activated areas including the right AI and left IFG, and cognitive self-reflection (implicating internal models) activated left IFG/AI and DMN areas such as the PCC, only the left IFG (BA 44,45,47) and mPFC were activated in both conditions (Herwig et al., 2010). Similarly, the feeling of familiarity elicited by stimuli of different modalities and referring to the participants’ life experience was associated with activation of the left hemisphere, specifically in the IFG (BA 47) and DMN areas including the precuneus, the angular gyrus, the parahippocampal gyrus, and the hippocampus (Plailly et al., 2007). By contrast, the feeling of unfamiliarity was related to a smaller activation pattern mainly located in the right AI and likely related to the detection of novelty.

### SUMMARY

It is important to distinguish between mental controls for handling novelty and unpredictable circumstances (i.e., reactive control) and mental controls for handling familiar and predictable circumstances (predictive control), both in brain function and behavior. While areas including the IFG and AI are examples of a larger reactive control system, the DMN, together with the dorsal executive system, are part of a larger predictive control system in PARCS theory. The right IFG is involved in appraisal and detects stimuli that require elaboration or scrutiny from reactive control. The left IFG takes control during reappraisal when elaboration or scrutiny is needed to ensure consistency of new information with internal models.

### COMPARISON WITH OTHER THEORIES

Because of the fundamental nature of PARCS in the brain, PARCS bears relations to other theories from disparate areas. In focusing on the DMN as part of the predictive control system, we discussed relations with theories of DMN function (see The Default Mode Network as Part of The Predictive System). Focusing on the AI as part of the reactive control system, we derived an interpretation of insula function and structure from PARCS that seems largely compatible with Craig’s (2009) theory. However, instead of focusing on the role of the AI in a type of awareness and dismissing the less integrated or more differentiated posterior insula as representing a lower-level processing stage, PARCS suggests a role of the posterior insula in different types (differentiated vs. undifferentiated) of awareness (see The Anterior Insula as Part of the Reactive System), and differential relations with anxiety. These theories are integrated with empirical support and with theories of continuous functional and connectivity gradients in the brain and how activity along those gradients can shift in functionally meaningful ways via corticostriatal loops. We have also integrated theories of hemispheric functional asymmetry based on cognitive, lesion, and split-brain research, pointing to the left IFG as providing elaboration when necessary in the communication between reactive and predictive processing (see Appraisal and Reappraisal in the Right and Left Inferior Frontal Gyrus). We also discussed similarities with Ranganath and Ritchey’s (2012; see The Anterior Insula as Part of the Reactive System) memory model. One of PARCS’ strength is that it binds together these previous theories (and/or the phenomena they explain) in a meaningful way.

At this stage it is also prudent to explain some of our terminologies, in particular in terms of contrasting PARCS and the dual mechanisms of control (DMC) framework by Braver and colleagues (see Braver, 2012). This is important, as we initially used the same labels of “reactive control” and “proactive control” that Braver (2012) used. Potential confusion that may arise in relation to their model was the reason why more recently we switched to using the labels reactive and *predictive* control. Moreover, earlier we have mentioned (Tops and Boksem, 2011a) that most of the control that is called proactive control in their DMC framework actually is part of reactive control in PARCS (the kind of tasks employed in their studies typically do not involve predictive control). For instance, their proactive control reflects the active maintenance of task goals associated with sustained and/or anticipatory activation of lateral PFC, which does not need to involve predictive or feedforward action models but merely the maintenance of working memory in the IFG, classifying as reactive control in PARCS theory. Moreover, as we will further explicate below, control by the reactive system is also not purely reactive in the sense that it includes tasks like anticipatory vigilance and associative short-term prediction. This latter idea explains why both DMC’s reactive and proactive control are mediated by comparable (often reactive/ventral system) brain areas, but predictive control in PARCS is not.

Other theories that deserve discussion of their comparison with PARCS are the one by Tucker and colleagues (Tucker et al., 1995, 2003; Tucker and Luu, 2007) that provided the basic departure point for PARCS, and that by Menon and Uddin (2010) and Corbetta et al. (2008) that describe dorsal and ventral cortical systems and shifts between them. However, those theories focus on attention and cognitive task performance and do not include functions in motivation and emotion [with the notable exception of Tucker and colleagues (e.g., Tucker et al., 1995, 2003)], have no functional interpretation for hemispheric asymmetry or the role of left ventrolateral cortical areas, and are mostly silent regarding different types of awareness and internally directed cognition. For this reason, detailed comparisons with those theories would be out of place in the present paper and are reserved for future discussions focusing on consequences of PARCS for externally directed cognition during cognitive response tasks. An important advantage of PARCS theory is that it provides predictions, interpretations, explanations, and organizing structure in areas outside cognitive task control, such as in the areas of personality and psychopathology (Tops et al., 2010), attachment theory, oxytocin function, and addiction (Tops et al., 2014a), effort, stress, and cortisol regulation (Tops et al., 2013c, 2014b) and internally directed cognition.

We will limit ourselves here to just one, global-level comparison. Cognitive control theories tend to focus on the functions of the dorsal ACC (i.e., anterior midcingulate cortex) and/or mPFC, and when areas such as IFG or AI are addressed, they typically are not implicated in equally important control functions (e.g., Botvinick et al., 2004; Shackman et al., 2011). In PARCS theory, the right IFG is a cognitive control area of the *reactive* control system that responds to novelty and salient stimuli, but is also implicated in, for example, maintaining stimuli in a working memory buffer for sustained processing (i.e., sustained appraisal), and in sustained attention. Sustained attention can be very useful in unpredictable circumstances and does not involve predictive models, and is implicated in anticipatory anxiety. Neuroimaging evidence supports the implication of the right IFG in effortful sustained attention that prevents lapses and mind wandering. Several studies have suggested that increased attentional effort during performance over extended periods of time or after sleep deprivation is associated with increased activation of right-hemisphere ventral cortical areas including IFG/AI, and sometimes in the context of activity declines in dorsal ACC and/or DLPFC (for a review, see Tops et al., 2013c). Moreover, the compensatory recruitment of the right IFG/AI after lapses of attention (i.e., mind wandering) is associated with recovery from lapses in attention (Weissman et al., 2006).

Notably, the predictive processing framework, that applies to the predictive control system and internal models in PARCS, has generated considerable interest in recent years (Clark, 2013a,b; Seligman et al., 2013). According to Clark (2013a), the brain as an action-oriented prediction machine provides a unifying framework for perception, action, and cognition (and homeostatic control, we would add). However, Clark himself points out that the predictive processing framework fails to specify the overall form of a cognitive architecture. It fails to specify specific neural mechanisms that are employed in specific circumstances and how they are arranged to explain complex behavior (Rasmussen and Eliasmith, 2013). Moreover, it fails to specify mechanisms that are employed in unpredictable or novel circumstances. We do not pretend that PARCS in its present form provides answers to all relevant questions. Rather, we regard PARCS theory in its present form as a starting point, readily providing relative comprehensiveness and detail as a theory of human behavior and cognition as compared to related theories.

## THE DYNAMICS OF REACTIVE AND PREDICTIVE CONTROL SYSTEMS IN INTERNALLY DIRECTED COGNITION AND MINDFULNESS

In this section, we apply PARCS theory to explain aspects of internally directed cognition including self-awareness, mind wandering, rumination, mental imagery, autobiographical memory, and mindfulness. Specifically, Clark (2013a) suggested that predictive systems, building from their function to facilitate perception by providing prediction and context, develop the ability to self-generate mental imagery by driving perceptual areas in predicted patterns. Additionally, mental imagery may partly implicate similar brain networks as autobiographical memory. Daselaar et al. (2010) proposed that the DMN represents the core of the modality-independent imagery network. As discussed in Section “The Default Mode Network as Part of the Predictive System,” The DMN has been proposed to support an ability to perform internal mentation (Buckner and Carroll, 2007). However, DMN *and* IFG activation correlated with vividness of imagery, suggesting recruitment of reactive control areas as well (Zvyagintsev et al., 2013). For internally directed cognitions, both the reactive *and* predictive systems are thus likely to be recruited, and, as suggested by PARCS, such introspection may well form the basis of abstract cogitation related to concrete and contextualized experiences (cf. Barsalou, 2008).

The same processes may be activated during the reading of stories, which activates internal models of world knowledge and self-narratives, as well as mental imagery. Relating new experiences from a narrative to the self may implicate left IFG functions in searching, retrieving, and integrating world knowledge into linguistic representations and detecting world knowledge anomalies (Kuperberg et al., 2003; Hagoort et al., 2004). During story comprehension this area coactivates with the DMN implicated in self-related processing and mentalizing (Mar, 2011), producing a pattern of activation that is similar to other examples (given in Section “Appraisal and Reappraisal in the Right and Left Inferior Frontal Gyrus”) where new information had to be integrated with internal models.

We can imagine looking at ourselves (third person, observer perspective) or looking through our own eyes (first person, field perspective). Internally directed cognition and self-reflection can involve different spatial (e.g., field vs. observer) and temporal (e.g., momentary vs. retrospective vs. prospective) perspectives and different attentional modes (e.g., a mindfulness mode). Mind wandering typically involves imagery, episodic memory, and other self-reflective capacities that can vary in perspective and attentional mode. Characteristics such as perspective may help us understand which mechanisms are responsible for mental experience at any given moment. In the section that follows, we now discuss how PARCS can aid in understanding different forms of awareness and self-reference and in applying different spatial perspectives in internally directed cognition and mindfulness.

### THE RUMINATING AND THE WANDERING MIND

Mind wandering has been thought of as the engagement in cognition unrelated to the current demands of the external environment (Schooler et al., 2011). As such, the term mind wandering may not refer to a specific kind of internally directed cognition but instead may involve different kinds including reflection and self-awareness. Notably, research on individual differences in internally directed mental states such as self-consciousness suggests that two states can be discriminated that are in some regards very different but are nevertheless positively correlated and easily confounded: rumination and reflection (Trapnell and Campbell, 1999). They are different in their correlations with third variables, reflection being associated with more accurate and extensive self-knowledge and lower psychological distress whereas rumination is associated with higher psychological distress, social anxiety, depression, obsessive thinking, external control, self-discrepancy, and other-directedness (Watson et al., 1996; Trapnell and Campbell, 1999; Ben-Artzi and Hamburger, 2001–2002; Ghorbani et al., 2004; Takano and Tanno, 2009). We discussed the role of the DMN and predictive control in prospective reflection in Section “The Default Mode Network as Part of The Predictive System.” In Section “The Ruminating Mind,” we build on the role of the left IFG/AI in integrating novel information with internal models, to explain the role, mechanism and place of rumination in PARCS. Next, in Section “The Wandering Mind,” we show that the combination of mechanisms of reflection and rumination allows PARCS to describe the processes that take place during mind wandering.

Within PARCS, rumination reflects elaborative, often self-related, processing of incongruency between incoming information and internal models. Hence, PARCS predicts coactivation and/or functional connectivity between left IFG/AI and DMN areas involved in processing related to the self and internal models (see The Ruminating Mind). Mind wandering not only sometimes reflects rumination, but also, at other times, prospective reflection. Whereas during rumination, mind wandering is often concerned with problematic happenings from the past, social-evaluative concerns and negative affect, during reflection it is associated with prediction of successful actions and with positive affect. Prospective reflection involves predictive control system areas (e.g., DMN) without left IFG/AI involvement. Mind wandering does not involve and is negatively related to right hemisphere reactive control (see Comparison with Other Theories; except the specific case of sustained appraisal of salient stimuli if this is regarded mind wandering) and dorsal executive system activation.

#### The ruminating mind

Based on PARCS, we have now tried to clarify how the left IFG is important in the process of assimilation of novel and emotional information into existing internal models. Specifically, in Section “Appraisal and Reappraisal in the Right and Left Inferior Frontal Gyrus,” we have discussed how the left IFG reactive control is involved in reappraisal and elaborative processing when more automatic processing of predictive control cannot do the job. This elaborative processing may turn into rumination in the form of prolonged and repeated contemplation of problematic information, self-contemplation, and self-examination.

Certain people, especially those suffering from social anxiety and social evaluative concerns, even apply a distinctive type of rumination, involving reappraisal, but in the “wrong” direction: instead of making information acceptable for inclusion in internal models, by contrasting information with internal models of what is acceptable to others, the impact of social signals is intensified as part of a strategy to prevent rejection (Mikulincer and Shaver, 2007). This type of rumination does not solve problems but instead is related to prolonged and recurrent contemplation of social concerns, and often to unfavorable consequences. Moreover, the social concerns induce self-discrepancy, i.e., inconsistencies between internal models, triggering ruminative self-processing (Ben-Artzi and Hamburger, 2001–2002).

Consistent with overlapping functions in PARCS, the brain mechanisms behind this self-reflective kind of rumination are similar as those behind positive reappraisal and those behind an adaptive (problem-solving) type of rumination – which we will refer to as reflection from here on forward – because they involve comparable cognitive functions for elaborative self-reflection in the left IFG (for a review, see Trapnell and Campbell, 1999; Andrews and Thomson, 2009). Specifically, whereas reflection involves contextualization by activating internal models, anxious anticipation (i.e., appraisal-related rumination) has been found to activate the right AI, and more so in trait anxious individuals (Carlson et al., 2011; Simmons et al., 2011; see The Anterior Insula as Part of the Reactive System). What is the key difference for more chronic ruminative (vs. reflective) types of dealing with novel information can be found by examining trait anxious individuals, for whom the right AI anticipatory activation showed stronger connectivity to the left IFG and DMN areas including the precuneus and PCC. The latter finding may reflect the greater tendency of trait anxious individuals to engage in negative self-directed attention (Simmons et al., 2011).

Predictive and reactive control system predicts that elaborative self-reflection involves co-activation of the left IFG/AI with DMN areas involved in self- and other-reflection, perspective taking, internal models, and memory (Modinos et al., 2009). In turn, this means that a host of elaborative self-directed attention involve activation of the left IFG/AI and the DMN, such as trait anxiety, social anxiety, and self-focused rumination and depression. Consistent with this prediction, trait rumination predicts activation of left IFG (BA 47) during both observing negative images and when reappraising neutral images in a negative way, while in the last case there tends to be coactivation of DMN areas implicated in self-referential thought (Ray et al., 2005; cf. Engels et al., 2007). Furthermore, social anxiety patients showed less DMN deactivation during perception of neutral and emotional faces than controls, suggesting that sustained activation of the DMN reflected feeling of wariness of others’ judgment and self-focused rumination (Gentili et al., 2009). Finally, self-focused rumination in depression is also believed to involve persistent DMN activation (Lemogne et al., 2012).

If both the left IFG/AI and the DMN are involved in negative self-directed attention, then one would also expected increased functional connectivity between these areas during times of anxiety and rumination. Indeed, while reappraisal often efficiently resolves emotional issues, large incongruencies between novel information and internal models or inconsistencies and conflicts within or between models themselves may trigger self-reflective rumination, in the form of elaborative processing from the left IFG/AI that “hijacks” the DMN. Only recently the first studies have been published of the functional connectivity of the DMN or left IFG/AI during anxiety and rumination. These studies do suggest the hypothesized functional connectivity between the left ventral control areas and the DMN. Severity in major depression predicted functional connectivity between the left AI and the pregenual ACC that is part of the DMN, which may reflect increased self-related rumination (Horn et al., 2010). Similar results have been obtained in healthy subjects. Self-reported state anxiety during a resting-state scan related to increased connectivity of the left AI to the DMN (Dennis et al., 2011). In another study, after the end of a social stressor there was increased functional connectivity of the amygdalae with the PCC (Veer et al., 2010). Another study that separated AI activation during anxious rumination from activation during interoception and focal attention, found that whereas the last two activations lateralized to the right mid- and AI, anxious rumination was lateralized to the left AI (Simmons et al., 2013). Moreover, anxious rumination produced substantially more coactivation in DMN areas (including PCC, precuneus, left parahippocampal gyrus, and bilateral hippocampus) and selective functional connectivity to left (more than right) IFG, left (more than right) caudate, left precuneus and angular gyrus, and bilateral dorsomedial PFC and DLPFC.

In summary, the findings appear to be consistent with involvement in the ruminative process of co-activation of the left IFG/AI with DMN areas. This reactive control from the let IFG/AI hijacks areas that have access to internal models for elaborate processing.

#### The wandering mind

We have now focused on internally directed cognitions, such as rumination and reflection. Another important aspect of internally directed cognition is mind wandering. PARCS allows to discriminate between two types of mind wandering that very much run parallel to the modes of integrating novel information: (1) a prospective and reflective type associated with predictive control, related to planning, optimism, and DMN activation; (2) a ruminative type reflecting reactive control associated with a tendency to negative affect, problem solving or self-reflection and coactivation and/or functional connectivity between the left IFG and the DMN (see The Ruminating Mind).

Consistent with predictive control during a prospective type of mind wandering, recent research suggests that during mind wandering, consciousness becomes decoupled from perception, providing an opportunity to guide behavior using internally represented plans and goals, or internal models (Antrobus et al., 1966; Baumeister and Masicampo, 2010; Baumeister et al., 2011; Smallwood et al., 2013b). Reports of task-unrelated thoughts obtained during performance of non-demanding tasks support the hypothesis that off-task thought can be a process that aids preparation for future events: when thoughts are decoupled from current tasks, occurring thoughts are often internally and future-focused, taking the form of autobiographical planning (Baird et al., 2011). Neuroimaging studies support this functional interpretation by linking mind wandering to activation of the DMN (and thus decoupling of conscious thought from perception) that is believed to sub-serve functions of prospection, action planning, and simulation (see The Default Mode Network as Part of The Predictive System). Individual tendencies to mind wander away from the direct physical experience from pain have also been associated with internally directed DMN activation (Kucyi et al., 2013). These findings support our first suggested form of mind wandering, a prospective and reflective type, related to planning, optimism, and DMN activation.

Other findings are suggestive of reactive control during a more ruminative type of mind wandering. Although mind wandering typically involves future thinking to a significant extent, unhappy moods may lead to a retrospective bias in mind wandering (Smallwood and O’Connor, 2011), possibly reflecting depressive rumination aimed at trying to work out or solve things that have gone wrong before future actions are planned and initiated (Andrews and Thomson, 2009). Smallwood and Schooler (2006) have suggested that mind wandering may be a mode of problem solving. In particular, they suggested that mind wandering is a situation when controlled processing becomes hijacked in the service of current concerns, much similar to ruminative ways of dealing with current emotional states (see The Wandering Mind). If this is correct, then this type of mind wandering includes elaborative kinds of rumination processes that are associated with trying to solve problems that have, so far, eluded solution. This interpretation in PARCS would suggest the involvement of the left IFG in this ruminative process (see The Ruminating Mind). Consistent with this prediction, left IFG/AI coactivated together with DMN areas prior to self-reports of mind wandering (Christoff et al., 2009) and prior to self-reports of mind wandering or task-related interference, which was suggested to reflect executive processes involved in the management of personal goals and concerns (Stawarczyk et al., 2011).

Consistent with these two types of mind wandering, researchers have detected links to both positive (Mar et al., 2012) and negative emotion (Killingsworth and Gilbert, 2010; Smallwood and O’Connor, 2011). Smallwood et al. (2013b) explain the heterogeneous array of correlates of mind wandering by suggesting that experimental measures of task-unrelated thought might actually be confounding different types of experience. Although a small subset of fundamental processes may be common to all examples of task-unrelated thought (e.g., episodic memory that generates the mental content, and the decoupling of attention from perception that supports internal focus), there may well be psychological processes that discriminate between mind wandering experiences with different qualities. Smallwood et al. (2013b) suggest that mind wandering produces episodes that are productive (i.e., predictive) and other episodes of a ruminative type that can be disruptive; some might bring joy to the experiencer and others pain. PARCS provides the underlying mechanisms for different types of mind wandering and embedding them into a context of emotional control.

### THE SPATIAL AND TEMPORAL PERSPECTIVES OF REACTIVE AND PREDICTIVE CONTROL SYSTEMS

Predictive and reactive control system suggests ways in which predictive, reactive, and ruminative control are associated with different subjective qualities of awareness. Those differences can be experienced at different times during mind wandering, but also during introspection, imagery, and autobiographical recall. In this section, we not only discuss whether PARCS can explain phenomenology that is described in research on self-awareness, imagery, and autobiographical memory, but also discuss aspects of the control modes that are addressed in this literature and that can help discriminate the different control modes as they alternate over time during mind wandering or mindfulness training (see The Anterior Insula as Part of the Reactive System).

In (self-)awareness, it may be difficult to subjectively discriminate ruminative from predictive control types because elaboration or contemplation on awareness makes it the object of left reactive control (related to rumination). Moreover, both types of control have access to internal models, which renders them relatively contextualized and detached. Indeed, in Section “The Wandering Mind,” we discriminated ruminative and prospective mind wandering purely on the basis of different (and sometimes even opposite) correlates. Given low subjective discrimination between ruminative and predictive control types, PARCS suggests that descriptions of self-awareness contrast the right hemisphere reactive control type (characterized by vivid experience of the here and now and arousability) from a type that may reflect ruminative or predictive control (including characteristics such as self-reflective, contextualization and relative detachment from the here and now and hence decreased arousability; see The Reactive vs. the Predictive Self). Those two types of self-awareness are further characterized by a field vs. observer perspective, respectively^2^, that have similar correlates as the respective types of self-awareness and that are recognized or manipulated in studies of imagery and autobiographical memory (see Field and Observer Perspective in Imagery and Autobiographical Memory).

#### The reactive vs. the predictive self

In terms of the neural correlates of consciousness, PARCS integrates and connects two competing theories. The DMN has been suggested as a candidate for the network subserving basic functions related to consciousness (Boly et al., 2008; Greicius et al., 2008). Indeed, DMN connectivity is decreased in severely brain-damaged patients, in proportion to their degree of consciousness impairment (Vanhaudenhuyse et al., 2010). However, activity in this area is inversely correlated in functional-imaging studies with the activation in the AI that is associated with awareness and task-related attention (Craig, 2009). Moreover, the right hemisphere reactive orienting/appraisal system closely corresponds to the set of cortical regions damaged in patients with hemineglect syndromes in whom unpredicted sudden stimulus changes do not enter awareness (see Downar et al., 2000). This has led to competing theories of the neural substrates of awareness, stressing either the role of the DMN in self-awareness extending across time or the role of interoceptive and emotional information integration in the AI in subjective feeling and awareness of the emotional moment. By contrast, PARCS seeks to integrate these different aspects or qualities of awareness in one framework by relating them to predictive (associated with internal models) and reactive control, respectively.

Predictive and reactive control system predicts two fundamentally different modes of self experience, the experiential (reactive) mode of the right predictive system that is focused on context-free momentary experience, and experience in the context of predictive system internal models that include past, present and future. As we have discussed, during self-elaboration, the left IFG may connect aspects of the reactive mode to the predictive mode of cognition, also implicating internal models. However, the processing modes of the right reactive system and the controls involving internal models appear to be two extremes that contrast most subjectively striking with each other, which may be recognizable in different modes of phenomenal awareness and the self. Descriptions in the literature of different forms of awareness and self-reference appear to match the description of the right reactive system control and control involving internal models. Here, we will discuss how these different control systems can be involved in different forms of self-awareness and self-reference.

Many scholars have suggested that there are two facets of the self – one defined by experiential awareness and the other by conceptual knowledge (Damasio, 1994; Gallagher, 2000; LeDoux, 2002; Wilson, 2002; Epstein, 2003). This corresponds with the experiential (i.e., context-free, appraising) right reactive mode and the internal model (predictive as well as the left reactive mode working with those models), respectively. James’ (1890/1950) notion of the “I” (self as experiencer) vs. the “me” (self as object) is a well-known example of this distinction. The experiential “I” self can be understood as emerging in a bottom-up fashion, evoked by concrete momentary features of the environment and one’s actions on it (right reactive mode). The conceptual “me” self reflects internal models of the self as an abstract entity that spans across time (internal model mode). These internal models that guide the experience of the self have been described in terms of diverse meaning structures including self-schemas (Markus, 1977), self-theories (Ross, 1989; Hong et al., 1999), and self-narratives (Singer and Salovey, 1993; Neisser, 1994; McAdams, 2001).

In a similar distinction showing an internal model mode and a right reactive mode, extended self-reference links experiences across time (internal model mode), whereas momentary self-reference is centered on the present (right reactive mode; Farb et al., 2007; Craig, 2009). It has been suggested that the experiential “I” represents a more primitive awareness of self that we may share with other animals, whereas the conceptual “me” is an elaborated version relying on uniquely human capabilities for language and self-reflection (Damasio, 1994; Gallagher, 2000; Farb et al., 2007). For example, Gallagher (2000) proposed two important concepts of self: the “minimal self,” a self devoid of temporal extension, and the “narrative self,” which involves personal identity and continuity across time. Gallagher relates the narrative self to Gazzaniga’s left-hemisphere “interpreter” and episodic memory (see Appraisal and Reappraisal in the Right and Left Inferior Frontal Gyrus). The minimal self may reflect a minimal form of self-consciousness, namely pre-reflective self-consciousness, which was argued to be a constant structural feature of conscious experience that corresponds to the consciousness of the self-as-subject that is not taken as an intentional object (i.e., right reactive experiential mode; Legrand, 2007).

It has been suggested that the two aspects of the self are supported by different neural systems (Gallagher, 2000; Northoff and Bermpohl, 2004). For instance, emotional and interoceptive signal processing in the posterior insula has been related to bodily self-consciousness and the “I” (Heydrich and Blanke, 2013; Park and Tallon-Baudry, 2014). However, activation in the right AI, ventromedial PFC and the precuneus are functionally associated for accessing interoceptive information and underpinning subjective experience of the emotional state (Terasawa et al., 2013). Thus, awareness of one’s own emotional state appears to involve the integration of interoceptive information with an interpretation of the current situation derived from internal models (see **Figure 2**). In the following section, we explore how the influences from reactive and predictive control on self-processing and memory may result in the adoption of different perspectives in imagery and autobiographical memory.

#### Field and observer perspective in imagery and autobiographical memory

Predictive and reactive control system integrates the implication of the DMN in internally directed cognition and self-reflection with a role for the left IFG in the case of (self-)rumination. Moreover, the right reactive system is involved in an experiential, context-free direct field perspective of being in the world, while the left reactive control is involved in rumination and problem solving, suggesting a more observing perspective. By contrast, predictive control is flexible in terms of perspectives that can be of the self, other, observer or field (in this case the field perspective may involve coactivation or functional connectivity between the DMN and executive parts of the predictive system). In terms of recall, people can recollect an event as if they were seeing it again through their own eyes (field) or from the perspective of a detached spectator (observer). We will discuss evidence that experience that is experiential (e.g., focused on feelings and often reflecting the right reactive mode) tends to adopt a field perspective, while experience in an analytic or reappraising (left reactive) mode adopts an observer perspective.

Consistent with the context-free right reactive mode, picturing an event from the field perspective involves a bottom-up style of constructing meaning in which people incorporate information about the experience evoked by concrete features of the pictured situation and define the event in terms of these constituent aspects. Consistent with the left reactive mode, picturing an event from the observer perspective involves a top-down style of meaning making in which people integrate a pictured event within a broader context and define the event in terms of the abstract meaning that results (Libby and Eibach, 2011). Individuals experimentally induced to focus on their feelings about an experience are more likely to recall the event from the field perspective, whereas individuals induced to focus on the objective circumstances of an experience are more likely to recall the event from the observer perspective (Nigro and Neisser, 1983).

Indicative of a decreased experiential mode (decreased appraisal and right reactive control), memories naturally retrieved from the observer perspective are related to less reliving, fewer visual images, less sensory information, less urgency, and less certainty that the event occurred as the individual remembers it (Berntsen and Rubin, 2006). The distance provided by the observer perspective may be a functional part of left reactive control that allows the individual to more objectively observe the situation and subsequently reappraise, work through, and ultimately leave an emotional experience behind them. It may be easier to engage in this process of reappraisal or rumination when individuals can detach themselves from the direct painful or emotional experience (Wilson and Ross, 2003). Rumination and emotional detachment related to an observer perspective in depression (Williams and Moulds, 2007). Shifting the recall of a distressing intrusive memory of a negative autobiographical event by mildly dysphoric participants from a field to an observer perspective resulted in decreased experiential ratings: specifically, reduced distress and vividness (Williams and Moulds, 2008). Imagery of positive scripts produced more positive affect after field than observer imagery (Holmes et al., 2008).

When individuals adopt a wider, contextualized (“global” or “detached”) perspective, they activate predictive system areas. For instance, subjects who were instructed to view neutral and aversive social scenes as though they were an anthropologist viewing the scene objectively or an emergency room doctor maintaining a detached clinical perspective so that he can function coolly in the situation, they activated typical predictive system (DMN) areas such as the precuneus, PCC, middle temporal and angular gyrus and mPFC (Koenigsberg et al., 2010). Moreover, while distancing from aversive scenes, they coactivated the left IFG/AI (BA 47/45/13) together with the DMN areas and deactivated the amygdala, consistent with an observer view during reappraisal or rumination.

There are few more direct studies of field and observer perspectives. One study found significant decreases in bilateral insula and left somato-motor activity during the recall of observer memories, suggesting reduction in one’s cortical representations of the physical, embodied self when an observer perspective is taken (Eich et al., 2009). Additionally, there was a small relative increase in right amygdala activity coincident with the recall of field memories, providing limited support for right reactive system activation. In another study analyzing two independent datasets, the spontaneous tendency to recall memories from a field perspective was positively correlated with the volume of the anterior part of the right precuneus (Freton et al., 2014). Activation of the right precuneus suggests that this activation reflect the field perspective that can be adopted in the predictive control mode. Of note the right IFG and precuneus coactivate during autobiographical recall or imagery (e.g., Zvyagintsev et al., 2013), which may reflect context from internal models supporting perception and recall in the right reactive system (**Figure 2**).

Taken together, these findings suggest that a field perspective may reflect the right hemisphere reactive (experiential) mode. By contrast, making meaning of experience and integrating it with a broader context during rumination and reappraisal (left reactive) is performed from the observer perspective. By contrast, predictive control can adopt either a field or observer perspective. However, the sparse direct neuroimaging evidence is as yet inconclusive.

### MINDFULNESS

Although people have problems with subjectively discriminating the ruminative reactive (observer perspective) from the predictive control type of self-awareness, the predictive control type should actually be flexible in perspective taking, being able to take the perspective of self, others, observer and the field perspective. This ability of predictive control to take a field perspective that contrasts with the observer perspective of ruminative reactive control enables people to discriminate and experience awareness that purely relates to predictive control, through training techniques such as mindfulness meditation. Mindfulness meditation facilitates predictive system control of awareness by stimulating a field perspective through focusing on the here and now, preventing rumination, sustained appraisal, habitual behavior, prospection, and mind wandering. Despite this instrumental focus on the here and now, PARCS predicts that this type of predictive control (mindful) awareness should be characterized by detached observation, awareness of context, conscious access to the rich features of each experience, an autobiographical sense of identity that projects back into the past and forward into the future, and enhanced metacognition and self-regulation skills (i.e., availability of internal models).

Mindfulness meditation is increasingly included in therapies and interventions to boost resilience. PARCS theory suggests that, comparable to the cultivation of certain kinds of positive affect and emotion regulation, mindfulness meditation may increase resilience by inducing a shift from reactive control toward internal model-guided control (Tops et al., 2013a). We now discuss whether mindfulness features the characteristics of field perspective predictive system control as predicted by PARCS theory: detached observation, decreased appraisal, availability of internal models, and focus on the here and now.

#### Detached observation and increased self-observation skills

The mindfulness approach promotes detached observation, which presumably has the effect of increasing the individual’s capacity to tolerate difficult emotions. The accompanying exposure, possibly by assimilation of the emotional material in internal models, transforms such emotions into innocuous states (Kent and Davis, 2010). Mindfulness practice may result in improved self-observation skills, which may lead to better recognition of sensations, cognitions, and emotional states and improved ability to respond skillfully to these phenomena as they arise (Baer et al., 2004). Such “internal state awareness” has been described as the reflective type of self-consciousness that contrasts with the ruminative type by showing negative instead of positive correlations with social anxiety, depression, psychological distress, obsessive thinking, external control and other-directedness, and more accurate and extensive self-knowledge (Watson et al., 1996; Trapnell and Campbell, 1999; Ghorbani et al., 2004; Takano and Tanno, 2009). Increased self-observation skills may reflect the availability of predictive internal models to guide perception (**Figure 2**) and, relatedly, access to differentiated viscerosensory interoceptive representations in the caudal–dorsal insula (see The Anterior Insula as Part of the Reactive System). Indeed, in a recent study mindfulness meditation practice compliance predicted greater posterior (caudal–dorsal) insula and reduced visual pathway recruitment during interoceptive attention to visceral bodily sensations (Farb et al., 2012).

Demonstrating limitations to self-observation skills, people’s beliefs about who they are do not always correspond with the reactions that the environment evokes in them (cf. Nisbett and Wilson, 1977). PARCS suggests that this may be the case because effortful contemplation and rumination as directed by the left IFG from the observer perspective often implicates concerns about social rules and evaluation, and has no access to direct experience from the field perspective. Moreover, as discussed above, especially the predictive control system’s mode of contextualized perception and awareness may be difficult to contemplate. One way to gage insight into experience is to assess the correspondence of explicit reports with implicit measures. Mindfulness meditation and instructions to focus on one’s gut reactions to stimuli have both been shown to bring explicit attitudes in line with implicit measures (Gawronski and LeBel, 2008; Koole et al., 2009). Both of these manipulations share a conceptual similarity with the style of meaning-making that occurs with field perspective imagery, where people focus on their responses to the imagined environment, without judgment shaped by contemplation on their broader concept of the self. Indeed, findings reviewed by Libby and Eibach (2011) suggest that field perspective imagery has an analogous effect on the correspondence between explicit reports and implicit measures.

As an example of improved self-observation skills, mindfulness-based stress reduction therapy in social anxiety patients attenuated maladaptive habitual self-views by facilitating recruitment of the left IFG (BA 47) together with DMN areas in response to words describing negative traits during a self-evaluation task (Goldin et al., 2012). The implicit (uninstructed) nature of the task with regard to emotion regulation or rumination and short inter-stimulus intervals suggest that the change after mindfulness-based stress reduction therapy, which related to reduced anxiety symptoms, reflected reappraisal of habitual self-views (Goldin et al., 2012) which may have increased access to internal models and more differentiated and optimistic self-views.

#### Decreased appraisal

Reactive tendencies to extensively appraise, contemplate, inhibit, or otherwise to avoid sensations are prevented by increased capacity for tolerance and cool awareness (Kent and Davis, 2010). Non-reactivity scores from a trait mindfulness scale correlated negatively with rumination and negative bias and predicted reducing automatic emotional responding to negative stimuli via the insula after mindful breathing (Paul et al., 2013).

#### Availability of internal models

Consistent with the availability of internal models, mindfulness meditation is reflexive and goes with conscious access to the differentiated, rich features of each experience and enhanced metacognition and self-regulation skills (Lutz et al., 2008). Awareness of context and of the whole range of choices available (i.e., availability of internal models) at any given moment is increased. Mindfulness practice allegedly leads one to a clear but less emotionally reactive awareness of the autobiographical sense of identity that projects back into the past and forward into the future (Lutz et al., 2008; Kent and Davis, 2010). In other words, it appears that the availability and guidance by internal models is increased, thereby decreasing pure reactivity, and increasing resilience.

#### Focus on the here and now

In contrast to the open monitoring style of meditation such as mindfulness, the focused attention/concentrative style of meditation, used in the training of mindfulness skills, entails the capacities for monitoring the focus of attention and detecting distraction, disengaging attention from the source of distraction, and redirecting and engaging attention on the intended object (Lutz et al., 2008). We suggest that these are typical right IFG/AI functions in sustained attention (see Comparison with Other Theories). In both focused attention and open monitoring meditation, there is focus on the moment, which may function to prevent mind wandering and obtain a field perspective by preventing reactive system involvement in sustained appraisal of a stimulus, elaboration, inhibition, and rumination, and dorsal striatal involvement in habitual behavior. Both practices may increase skills in adopting a field perspective in the predictive system mode. *Acting with awareness* is a component of mindfulness that is contrasted with the concept of “automatic pilot” or habitual behavior in which behaviors occur without awareness because attention is focused elsewhere (Baer et al., 2004).

Supportive of an association between mindfulness and the field perspective are results in patients with a history of recurrent depression, where the tendency to retrieve observer perspective memories was associated with lower dispositional mindfulness (and with greater negative self-evaluation and greater use of avoidance; Kuyken and Moulds, 2009).

Although neuroimaging research on meditation is complicated by individual differences in strategies and non-linear effects of proficiency (Lutz et al., 2008), there is support for involvement of reactive system areas in focused attention meditation and of predictive system areas in open monitoring meditation. For instance, the most consistent effect in a study of practiced novices and expert Buddhist monks was the deactivation of the precuneus or PCC during focused attention meditation in contrast to activation of these areas during open monitoring meditation (Manna et al., 2010). In a PET study in which participants rated the level of relaxation (~open monitoring) and attentional absorption (~focused attention) during hypnosis, absorption-related activity was maximal in areas of the right reactive system: the right IFG (BA 45/47), the AI and the right inferior parietal lobule (Rainville et al., 2002). The IFG (BA 44) also displayed strong coactivation with absorption related thalamic activity. Absorption-related deactivations were found in lateral parietal cortices and in the precuneus. In another study, participants were scanned while they adopted either a reflective, extended self-reference that links experiences across time in memory (which may involve the predictive system) or a momentary experiential self-reference centered on the present moment (possibly reactive system). The experiential focus yielded reduced activity in predictive system areas such as mPFC, PCC, and hippocampus, and increased engagement of reactive system areas such as in the IFG, insula, and inferior parietal lobule (Farb et al., 2007).

The role of the right IFG in establishing, maintaining, and perhaps training skills in focused attention is illustrated in a recent study investigated mind wandering and attention during focused meditation by experienced practitioners (Hasenkamp and Barsalou, 2012; Hasenkamp et al., 2012). Using subjective input (e.g., participants pressed a button whenever they realized their mind had wandered) to investigate different phases of attention and mind wandering, the researchers found increased DMN activation during mind wandering (consistent with Section “The Wandering Mind”), while detection of mind wandering was associated with bilateral IFG/AI (BA 47/13) and dorsal ACC activation (consistent with those areas detecting the need for effortful reactive system intervention; see Appraisal and Reappraisal in the Right and Left Inferior Frontal Gyrus). After switching back to focus, during subsequent maintenance of attentional focus (which may involve right IFG especially in demanding conditions; see Comparison with Other Theories), there was increased connectivity between right dorsolateral PFC and right IFG/AI (BA 47/13). Individual differences in lifetime meditation experience correlated negatively with activation of areas including the right IFG/AI (BA47/13) and left IFG (BA45) during switching from mind wandering to focus, which suggests higher proficiency associated with decreased effortfulness. As noticed by Hasenkamp and Barsalou (2012) and Hasenkamp et al. (2012), the activation of right IFG/AI during detection, switching and maintenance may reflect this area’s role in switching between attentional networks (for instance when during predictive control a return to reactive control is needed; see Appraisal and Reappraisal in the Right and Left Inferior Frontal Gyrus).

Some studies found that training in mindfulness can reduce activation of the DMN (Brefczynski-Lewis et al., 2007; Tang et al., 2009; Brewer et al., 2011). However, Brewer et al. (2011) additionally found increased functional connectivity in experienced meditators between the PCC, dorsal ACC, and dorsolateral PFC, i.e., between the reflective and executive parts of the predictive control system (for similar results, see Taylor et al., 2013). Decreased DMN activation of the DMN but increased connectivity with the executive parts of the predictive control system may reflect present-moment field perspective (executive) awareness from the predictive system at the exclusion of mind wandering that is due to distraction from self-reflection and prospection.

A review of the literature extracted four components that seem to make up mindfulness, labeled “self-observation skills,” “acting with awareness,” “describing” (or “labeling”), and “accepting without judgment” (Baer et al., 2004). We discussed the first two components and interpret the second two components as indicating implicit, effortless and non-distracting strategies (i.e., without interventions in the form of effortful reappraisal or rumination) for the integration of experience with internal models, which would involve the left IFG.

#### Labeling

Labeling is the identifying, naming, and linguistic processing of the emotions that arise in a certain situation (Lieberman et al., 2007). Different studies showed that labeling, much like reappraisal, results in decreased physiological arousal, decreased activity in the amygdala and increased activity in left IFG including Broca’s area, and in the right IFG (Torrisi et al., 2013). Studies of emotion labeling of emotional prosody showed that implicit appraisal of prosody recruits the right or bilateral IFG, while explicit labeling requires the left IFG (Bach et al., 2008; Frühholz et al., 2012). Similarly, if not accompanied by rumination (“think about yourself, reflect who you are, about your goals, etc.,” activating left IFG/AI BA 45/47/13, the amygdala, and DMN areas PCC/precuneus), a self-referential mental state of making the actual emotional state aware (“feel yourself, be aware about your current emotions and bodily feelings,” activating the left IFG BA 44), is capable of decreasing amygdala activation and attenuating emotional arousal (Herwig et al., 2010). Brief, covert labeling of observed experience, using words or short phrases, such as “aching,” “sadness,” or “wanting to move” is often encouraged in mindfulness training. Notably, dispositional mindfulness has been associated with increased bilateral IFG (BA 47) and left insula activation and reduced bilateral amygdala activity during an affect labeling task (Creswell et al., 2007).

Further, during affect labeling, strong negative associations were found between the right IFG (BA 47) and right amygdala responses in participants high in mindfulness (Creswell et al., 2007), perhaps reflecting control of appraisal by the reactive system. Similar to the right reactive system activation during focused attention meditation, this activation may be instrumental in learning or achieving mindfulness, by preventing effortful (re)appraisal, rumination, or distraction. Dynamic causal modeling suggested that during affect labeling people in general show dampening influences from the right IFG toward the amygdala, as well as from the left IFG Broca’s area toward both the right IFG and amygdala (Torrisi et al., 2013). This dampening influence of the left IFG on right hemisphere reactive system areas may be implicated in the decreased arousal following linguistic processing, both in the observer perspective and during predictive system control.

#### Accepting without judgment

To accept without judgment is to refrain from applying evaluative labels such as good/bad, right/wrong, or worthwhile/worthless and to allow reality to be as it is without attempts to avoid, escape, or change it. This would facilitate integration of the experience with internal models without interventions in the form of effortful reappraisal or rumination. Indeed, left IFG activation in anticipation of uncontrollable pain related to less trait denial coping (e.g., “I pretend that it hasn’t really happened”) and more acceptance coping (e.g., “I learn to live with it”), suggesting inclusion in internal models instead of avoiding the processing of aversive experience (Salomons et al., 2007).

To summarize, PARCS provides a framework for understanding characteristics and consequences of mindfulness, the processes that facilitate mindfulness, and the accompanying brain activations. Mindfulness meditation facilitates a specific predictive system control mode of awareness by stimulating a field perspective through focusing on the here and now, which makes awareness in predictive control easier to discriminate and separate from the ruminative state which adopts an observer perspective. To obtain this mode of control, several techniques are used to prevent rumination, sustained appraisal, habitual behavior, prospection, and mind wandering. Through this undistracted predictive control system mode of awareness, mindfulness increases resilience, conscious access to the rich features of each experience, an autobiographical sense of identity that projects back into the past and forward into the future, and enhanced metacognition and self-regulation skills (i.e., availability of internal models to support perception and action).

**FIGURE 3:**
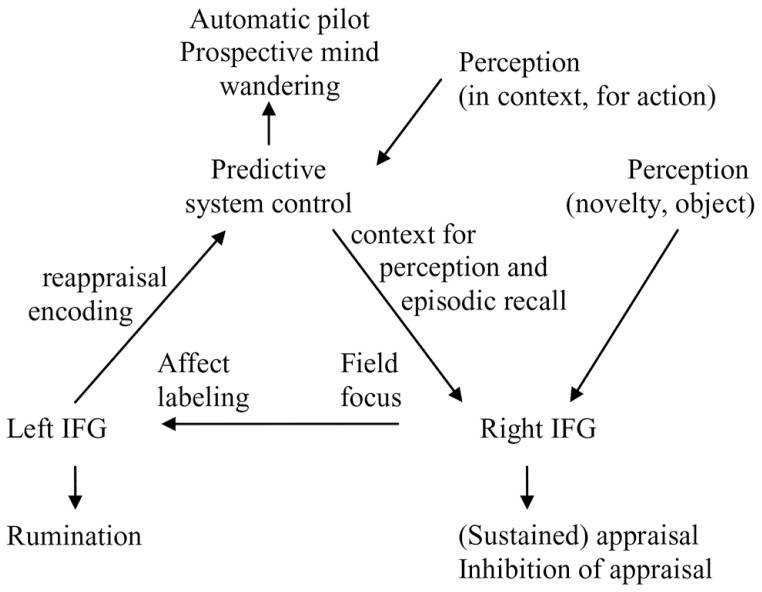
**Mindfulness as a natural flow of information processing (triangle in the figure), unobstructed by interrupting processes (outward arrows) of sustained appraisal or excessive inhibition of appraisal, rumination, prospective mind wandering, unconscious habitual behavior**.

According to PARCS, prospection through predictive control facilitates positive outcomes (see The Wandering Mind). Hence, future research should determine whether positive consequences of mindfulness meditation generalize to prospective mentation. Perhaps the focus on the here and now during mindfulness meditation only functions to facilitate a predictive system control mode or to prevent sustained appraisal, habitual behavior, and especially rumination that is difficult to discriminate from prospection. Another open question is whether the mindful state can involve coactivation or functional connectivity with components of the right hemisphere reactive system, generating a state in which the availability of internal models produces a mindful quality to appraisal. This last possibility could be part of a natural flow of information processing, unobstructed by interrupting processes (**Figure 3**).

The direction of flow of information in **Figure 3** is certainly not exclusive, but illustrates the natural direction from appraisal of novelty, to reappraisal and inclusion in internal models, to internal models guiding and increasing the richness of perception. The switch from right IFG to the verbal left IFG is facilitated by labeling of experience (Torrisi et al., 2013). Next, the left IFG facilitates intake in internal models (Perlovsky and Ilin, 2013) and is biased toward encoding (Tulving et al., 1994; Habib et al., 2003). Subsequently, coactivation of internal model areas with right IFG is associated with vivid experience (e.g., in imagery; Zvyagintsev et al., 2013) and there is a bias toward episodic recall in right IFG (Tulving et al., 1994; Habib et al., 2003). The information from internal models provides context to perception in the right hemisphere, facilitating observing skills and the richness of experience (Rotenberg, 1994; Hirsh et al., 2013).

## DISCUSSION

In the present article, we have outlined how PARCS provides a framework to understand characteristics of internally directed cognition and self-reflection, such as different qualities of awareness, mind wandering, mindfulness, and viewpoints during episodic recall and imagery. These characteristics may help discriminating the different mental states. The shifts between reactive and predictive control are part of processes that enable performance learning, intake and integration of new information with internal models, habituation of arousal from orienting responses, and integrated experience of the here, now and novel in a continuous personal context that supports understanding.

We also discussed cognitive control functions of the IFG that were at the same time similar and differentiated between the hemispheres. In both hemispheres, the IFG takes over control and induces elaborative processing when more fluent feedforward or automated processing fails or seems inappropriate or risky. Differences between left and right IFG on the other hand, appear to reflect specialization in the left IFG in not only verbal communication between individuals, but also, perhaps more fundamentally, in communication between reactive experience and predictive internal models by supporting the integration of novel information in those models.

Throughout the present article, we hope we have demonstrated how PARCS theory provides a promising framework that can integrate and explain a host of cognitive functions along with their phenomenological correlates. PARCS suggests functions and ways to discriminate mental states. Furthermore, PARCS provides a new way of thinking about many mental states. For instance, rumination and prospective mind wandering are often portrayed as purely maladaptive mental states. By contrast, PARCS suggests that prospective mind wandering reflects predictive control function, while rumination sometimes reflects elaborative reappraisal that aims to facilitate future predictive control by resolving incongruence of information with internal models (Andrews and Thomson, 2009).

PARCS also provides a framework for neuroimaging studies that try to delineate the neural mechanisms of mental state dynamics. For instance, PARCS predicts overlapping activations during reappraisal compared to self-rumination. Additionally, the dynamics of mindfulness are perhaps best captured by functional connectivity between the DMN and executive subparts of the predictive system, and processes that facilitate experience in the here and now and simultaneous extended awareness provided by context from internal models.

Finally, on an applied level, we presented information that may be used to facilitate shifts between mental states, for instance in the context of psychotherapy or self-help. Making oneself aware of interoceptive or external signals is the first step for their reappraisal (Gross and John, 2003) and for psychotherapeutic interventions that direct awareness away from for instance painful feelings toward pleasant aspects. The distancing quality of an observer perspective may be optimal in phases of therapy when emotional information needs to be elaborated upon without the client becoming overwhelmed by emotions. By contrast, the experiential nature of the field perspective may support the recovery of emotional memories that are being avoided. Alternatively, when chronic and unproductive rumination appears to be the main problem, mindfulness training and a field perspective can help in achieving a rumination-free mind state.

## Conflict of Interest Statement

The authors declare that the research was conducted in the absence of any commercial or financial relationships that could be construed as a potential conflict of interest.

## Footnotes

1 We offer the hypothesis that the activation of the left IFG during resting state may be related to the relative left-frontal activation (as indicated by lower EEG alpha power) that is often observed during resting state. This left frontal activation has been associated with individual differences in approach motivation (e.g., Davidson, 1998), which may reflect predictive/feedforward action control. Indeed, the left frontal EEG activation has been hypothesized to reflect activation of mechanisms of reappraisal that facilitate approach (Harmon-Jones, 2004).

2 At high arousal, increased narrowing of attentional focus may induce an object-centered instead of first person perspective (Easterbrook, 1959; Tucker, 2001). However, we assume that this is not the typical experience in mental imagery and autobiographical memory.
